# Preparation of High-Ductility Cement-Calcined Coal-Gangue-Powder-Composite-Based Rapid Repair Material

**DOI:** 10.3390/ma16176049

**Published:** 2023-09-03

**Authors:** Biaokun Ren, Lijuan Chai, Yuanzhen Liu, Yangkai Wang

**Affiliations:** College of Civil Engineering, Taiyuan University of Technology, Taiyuan 030024, China; renbiaokun@163.com (B.R.); liuyuanzhen@tyut.edu.cn (Y.L.); 19581592960@163.com (Y.W.)

**Keywords:** calcined coal gangue powder, high ductility, rapid repair, tensile performance

## Abstract

Coal gangue is a kind of solid waste. A high-ductility cement-calcined coal-gangue-powder-composite-based rapid repair material (HD-RRM) was prepared by partially replacing cement with calcined coal gangue powder (CCGP) for achieving high ductility and rapid hardening and conforming to the strength requirements of pavement layers. First, the physical and chemical properties and the reactivity of the CCGP were investigated. Second, HD-RRM material was prepared, and its tensile performance characteristic parameters were investigated. Lastly, the hydration products and microstructure of HD-RRM were characterized through tests (e.g., non-evaporable water content, scanning electron microscopy (SEM), X-ray diffraction (XRD), and comprehensive thermogravimetric analysis and differential scanning calorimetry (TG-DSC)). As indicated by the experimental results, the CCGP with a particle size of 1250 mesh exhibited the maximum potential reactivity. The optimal mixing ratio for HD-RRM in the experiments comprised a water–cement ratio of 0.27, a sand–cement ratio of 0.3, a fiber volume fraction of 2%, a cement content of 70%, a CCGP content of 20%, a fly ash (FA) content of 10%, and a superplasticizer content of 0.1%. Using the abovementioned mix design, the prepared HD-RRM was endowed with a 6 h ultimate elongation of 2.75%, an ultimate tensile strength of 7.58 MPa, a compressive strength of 45.4 MPa, and an average crack width of 125.53 μm, which meets the requirements of repair materials and provides a design method for CCGP resource utilization and asphalt concrete road and bridge deck repair.

## 1. Introduction

The stacking of coal gangue not only occupies land resources, but may also bring collapses, landslides, spontaneous combustion, and other secondary disasters, seriously threatening the safety of human life [1]. As cement is one of the main sources of high energy consumption and carbon emissions in the building materials industry, finding a low-carbon cementitious material to replace cement and reducing cement usage can accelerate carbon peak action. The carbon emission from gangue powder in the process of calcination is much lower than that of cement in “two grinding and one burning”. Considering the disease caused by the poor tensile deformation ability of concrete paving layers on road and bridge decks and accelerating the action program of “Double Carbon”, calcined coal gangue powder can be used to prepare cementitious composites [2].

As indicated by existing research, CCGP is capable of partially replacing cement to produce cement-calcined coal-gangue-powder-based composite materials. Chen et al. [3] utilized CCGP to prepare cementitious materials by replacing 30% of cement clinker, and the compressive strength at 3 d ranged from 15.0 MPa to 20.0 MPa. Yang et al. [4] prepared cementitious materials by replacing 0~40% Portland cement with CCGP, which exhibited compressive strengths ranging from 31.0 MPa to 17.0 MPa at 3 d. Zhou et al. [5] prepared cementitious materials with a 3 d compressive strength of 19.54 MPa and flexural strength of 4.69 MPa by replacing 5% ordinary silicate cement with CCGP. However, the cement used in the abovementioned materials is cement clinker or silicate cement, which has a slow hydration rate and low early strength, and does not conform to the requirements of strength grade, high ductility, and fast hardening for concrete pavement layers on roads and bridges, such that the result cannot provide effective theoretical guidance for the practical application of CCGP in engineering.

High-ductility cement-based composite materials have been well employed in the repair of concrete pavement layers on roads and bridges [6,7,8,9]. Fang et al. [10] synthesized high-ductility cement-based composite materials using Nissan Colly polyvinyl alcohol (PVA) fibers and FA, such that a 28 d ultimate elongation of 1.8% to 2.35% was achieved. Guo et al. [11,12] produced high-ductility cement-based composite materials using domestic PVA fibers and FA, with a 28 d ultimate elongation of 0.50% to 5.00% and compressive strength of 18.0 MPa to 70.0 MPa. Nevertheless, the cementitious materials applied for the above-described high-ductility composite materials primarily covered Portland cement, which did not achieve rapid hardening in a few hours and cannot directly apply to the rapid repair of concrete road and bridge pavements.

Numerous studies [13,14] have adopted used sulfate aluminum cement (SAC) as a replacement for Portland cement to prepare fiber-reinforced SAC materials. Zhu et al. [15] prepared fiber-reinforced SAC materials using polypropylene (PP) fibers, SAC, ordinary Portland cement, FA, weathered soil, and limestone powder, such that a compressive strength of 20.0 MPa, an ultimate tensile strength of 2.6 MPa, an ultimate elongation of 5.7%, and a crack width ranging from 30 μm to 90 μm were achieved at 28 d. Feng et al. [16] prepared fiber-reinforced SAC materials using micro steel fibers, SAC, silica fume, and river sand, such that a compressive strength of 12.4 MPa to 19.5 MPa and flexural strength of 4.0 MPa to 7.1 MPa were achieved at 3 h. Lv et al. [17] prepared fiber-reinforced SAC materials using SAC, fine quartz sand, and polyethylene fibers, such that a compressive strength of 31.0 MPa to 45.0 MPa and an ultimate tensile strength of 4.00 MPa with an ultimate elongation of 0.75% were achieved. However, the existing fiber-reinforced SAC materials did not consider the required strength grade and prominent tensile deformation capacity for rapidly repairing concrete pavement layers on roads and bridges.

According to the provisions of the “Technical Specifications of Cement Concrete Pavement Maintenance for Highway” (JTJ073.1-2001 [18]), the tensile strength of the crack repair material is more than 5 MPa, the elongation at break reaches 2–5%, and the compressive strength must reach more than 70% of the design strength. According to the “Specification of Cement Concrete Pavement Design for Highway” (JTGD40-2011 [19]) the design compressive strength of cement concrete is required to be 35MPa for extra heavy traffic class pavements, so the compressive strength of the materials used for pavement repair should be more than 24.5 MPa. Existing materials research work has not achieved the road and bridge concrete pavement layer strength level, high ductility, and fast hardness requirements.

Following the above results, a cement-calcined coal-gangue-powder-composite-based rapid repair material was developed by partially replacing cement with CCGP, with the aim of achieving high ductility and rapid hardening and conforming to the strength requirements of pavement layers. The mix proportions (CCGP, SAC, FA, water-reducing agent, ordinary river sand, and domestic PVA fibers) were first optimized in this study to prepare high-ductility rapid repair materials (HD-RRMs). After the material was cured for 6 h, the material was endowed with a strength grade of no less than C40, an ultimate elongation of no less than 2%, and an average crack width of less than 200 μm. Second, the tensile performance characteristic parameters of HD-RRMs were investigated. Lastly, the macroscopic properties of HD-RRMs were microscopically characterized. The material mix proportions for the rapid repair of concrete pavement layers on roads and bridges were obtained, and a novel pathway was developed for the comprehensive utilization of solid waste coal gangue.

## 2. Materials and Methods

### 2.1. Raw Materials

**Figure 1:**
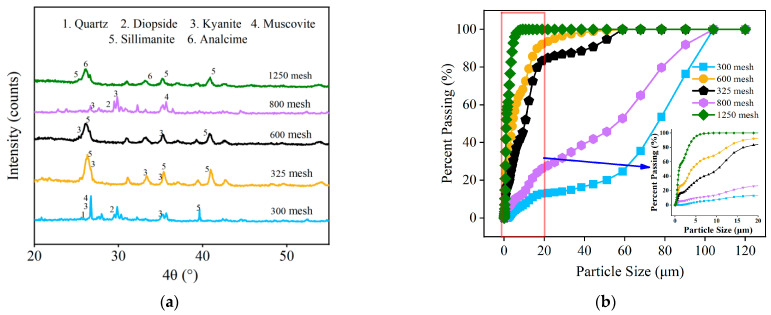

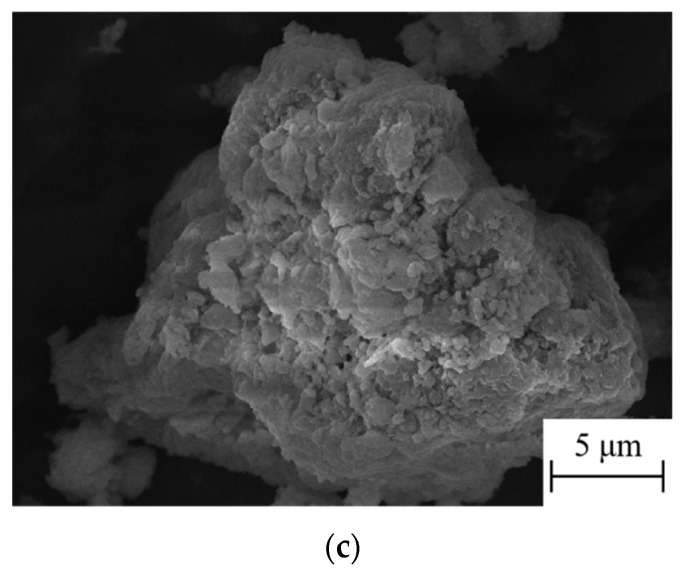
Microscopic testing of CCGP with different particle sizes: (**a**) XRD diagram, (**b**) cumulative particle size curve, (**c**) SEM micrograph.

Cement with a strength grade of 42.5 (SAC) was used, and its main performance indicators complied with the requirements of the standard GB20472-2006 [20]. The mineral admixtures included CCGP and FA. Different particle sizes of CCGP were selected, and their chemical compositions are provided in Table 1. Figure 1 presents the results of X-ray diffraction (XRD) analysis, particle size distribution curves, and scanning electron microscopy (SEM) images. Standard sand served as the fine aggregate for the reactivity testing of CCGP. During the preparation of HD-RRM, river sand with a maximum particle size of 1.18 mm and a fineness modulus of 1.0 was employed. Domestic PVA fibers were selected for reinforcement, with their properties listed in Table 2 [21]. The early strength agents applied comprised lithium carbonate (Li_2_CO_3_), sodium sulfate anhydrous (SSA), calcium chloride dihydrate (CCD), and a quick-setting agent mainly composed of alumina cement (QC). A powdered polycarboxylate superplasticizer was used as the water-reducing agent (PC), and tap water served as the mixing water.

### 2.2. Activity Testing of CCGP

The optimal particle size and replacement rate for CCGP were determined using a two-step activity testing method, referring to “Method of testing cements—Determination of strength” (GB/T 17671-2020 [22]) and “Fly ash used for cement and concrete” (GB/T 1596-2017 [23]):

(1) Particle size optimization: At a water–cement ratio of 0.5 and sand–cement ratio of 0.3, different particle sizes of CCGP were mixed with cement at a mass ratio of 3:7 to prepare mortar specimens. The specimens were cured for 6 h, 1 d, 3 d, 7 d, 14 d, and 28 d before their flexural and compressive strengths were examined.

(2) Replacement rate optimization: Based on the optimal particle size of CCGP determined in Step (1), the CCGP was partially substituted for cement at several replacement rates (e.g., 0%, 10%, 20%, 30%, 40%, 50%, 60%, 70%, 80%, and 90%). The specimens were cured under standard conditions and examined for flexural and compressive strengths at 6 h, 1 d, 3 d, 7 d, 14 d, and 28 d to determine the optimal replacement rate of CCGP. The dimensions of the strength test specimens were 40 mm × 40 mm × 160 mm.

### 2.3. Design Optimization of HD-RRMs

**Figure 2:**
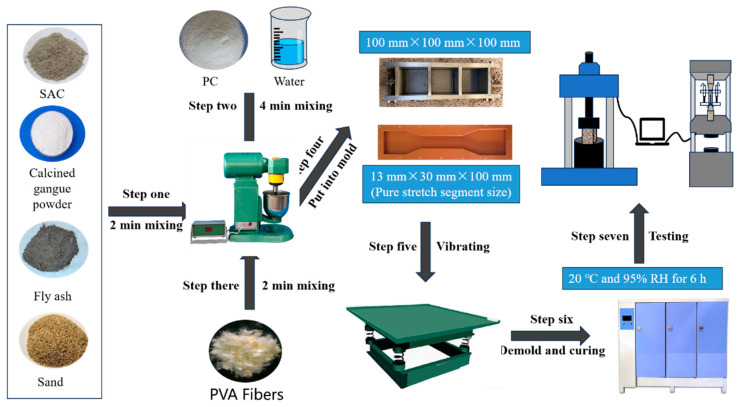
Flow chart of HD-RRM preparation.

Following the mechanical performance test method for high-ductility fiber-reinforced cement-based composite materials (JC/T 2461-2018 [24]), the design optimization of HD-RRMs was conducted in two steps:

(1) Based on the optimal particle size of CCGP determined in Section 2.2, cubic compression test specimens with dimensions of 100 mm × 100 mm × 100 mm were prepared by regulating the water–cement ratio, fiber content, early strength agents, and cementitious materials. The specimens were cured for 6 h, and their cubic compressive strength was examined to determine a mix proportion with a cubic compressive strength of no less than 40 MPa.
(2) In accordance with the mix proportions determined in Step (1), dog-bone-shaped tensile specimens were prepared, and their tensile performance was examined under standard curing conditions at 6 h. The mix proportions were optimized to achieve an ultimate elongation of no less than 0.5% and an average crack width of no more than 200 μm under uniaxial tensile loading. Figure 2 presents the flowchart for the preparation of HD-RRM.

#### 2.3.1. Design of the HD-RRM Mix Proportions

Table 3, Table 4, Table 5 and Table 6 list the preliminary mix proportions for HD-RRM (with cementitious material mass as 1, PVA fiber content as the volume fraction, and admixture content as a percentage of cementitious material mass).

#### 2.3.2. Mechanical Performance Test Methods

**Figure 3:**
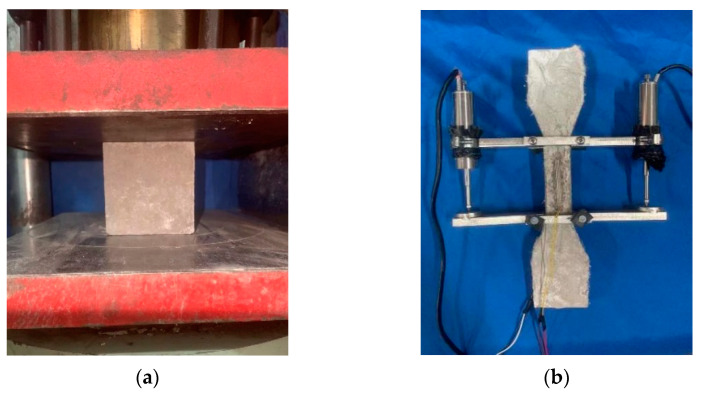
Test setup for HD-RRM mechanical properties: (**a**) compressive properties, (**b**) tensile properties.

The cubic compressive strength of HD-RRM was examined using a YAW-3000D electro-hydraulic servo pressure testing machine at a loading rate of 0.5 MPa/s. The stress–strain relationship under uniaxial tensile loading was examined using a universal testing machine at a loading rate of 0.002 mm/s for dog-bone-shaped specimens with strain gauges and displacement sensors attached in the central 100 mm zone. The data from strain gauges and displacement sensors were used to obtain the stress–strain relationship curve of HD-RRM. Figure 3 presents the apparatus for testing the compressive and tensile properties of HD-RRM specimens.

### 2.4. Microscopic Testing

The non-evaporable water content test method was adopted to represent the degree of hydration of cementitious HD-RRM. The hydration products of HD-RRM were detected and investigated using a Thermo Scientific Ultima IV XRD, Walthman, MA, USA, thermogravimetric analysis, and differential scanning calorimetry (TG-DSC). The microscopic morphology of HD-RRM was observed through a JSM-IT200 SEM.

## 3. Results and Analysis

### 3.1. Results of CCGP Activity Testing

#### 3.1.1. Effects of the Particle Size of CCGP on Mortar Strength

**Figure 4:**
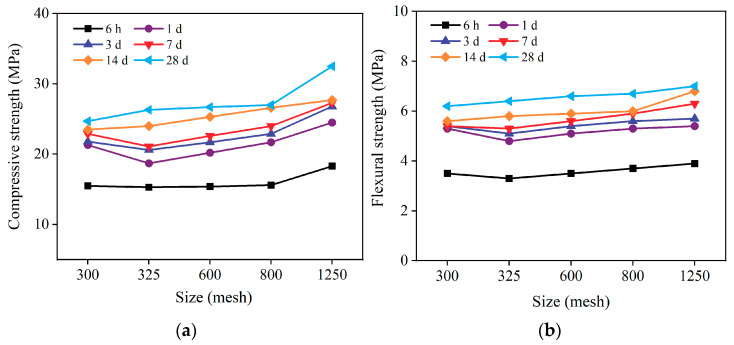
Strengths of cement sand test blocks with different CCGP particle sizes: (**a**) compressive strength, (**b**) flexural strength.

The compressive and flexural strengths of mortar specimens with different particle sizes of CCGP at various curing ages were investigated, as shown in Figure 4. In the standard curing age range of 6 h to 7 d, with an increase in the particle size of CCGP, compressive and flexural strengths of the mortar specimens exhibited a “valley” at 325 mesh, followed by an increase with further increments in particle size. In the curing age range of 14 d to 28 d, compressive and flexural strengths increased with an increase in the particle size of CCGP.

The 300-mesh CCGP contained more clinopyroxene (Figure 1a). Diopside contains 25.9% CaO, which can promote cement hydration and enhance early strength, resulting in higher strength for mortar specimens with 300-mesh CCGP compared with those with 325-mesh CCGP. Hence, the strength exhibited a “valley”. Particles in the range of 1 μm to 10 μm in CCGP are more conducive to its potential activity [25]. As depicted in Figure 1b, only the 1250-mesh CCGP had particles in the range of 1 μm to 10 μm. In the cementitious system, CCGP can serve as a physically compact filler while exhibiting higher specific surface area and better water absorption capacity with increasing mesh size. Consequently, it promotes faster hydration, thereby enhancing the early strength of the cement mortar specimens [5]. Thus, the mortar specimens with 1250-mesh CCGP exhibited higher strengths, and, based on the principle of maximum strength, 1250-mesh CCGP is preferred.

#### 3.1.2. Effects of CCGP Replacement Rate on Mortar Strength

**Figure 5:**
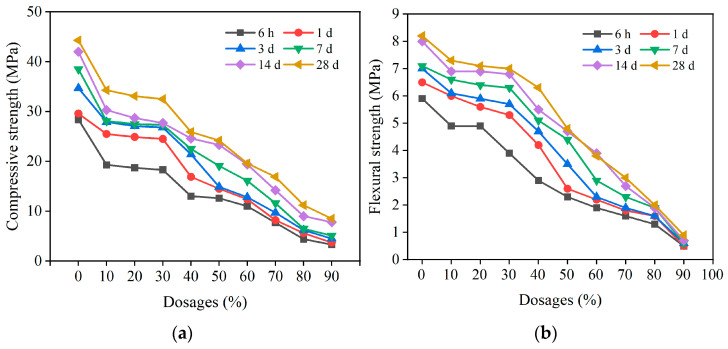
Strengths of cement sand test blocks with different CCGP replacement rates: (**a**) compressive strength, (**b**) flexural strength.

The compressive and flexural strengths of mortar specimens with different CCGP replacement rates at different curing ages were investigated (Figure 5). With identical curing ages, the cement mortar specimens with added CCGP exhibited lower strengths compared with pure cement mortar specimens; the greater the replacement rate of CCGP, the greater the reduction in strength was. The mortar specimens with CCGP replacement rates of 10%, 20%, and 30% achieved similar compressive strengths.

In the SAC-CCGP system, there were sophisticated interactions between cement hydration and the pozzolanic reaction of CCGP. On the one hand, the intensity of the pozzolanic reaction of CCGP was dependent on the concentration (pH value) of the CH solution, closely correlated with cement hydration. A higher concentration of the CH solution contributes to the pozzolanic reaction of CCGP [26]. As cement hydration progresses, CH concentration tends to increase, such that glassy SiO_2_ and Al_2_O_3_ in CCGP break down. As a result, active SiO_2_ and Al_2_O_3_ are released, and the pozzolanic reaction of CCGP can be facilitated. On the other hand, diluted CCGP cement was introduced, and the hydration products of cement were reduced. Moreover, with an increase in the replacement rate of CCGP, the effect of dilution on cement turned out to be more significant, such that the strength of the cement mortar was reduced. The compressive strengths of the mortar specimens with CCGP replacement rates of 10%, 20%, and 30% were not significantly different. The possible reasons for the above result include a balance between the dilution effect on cement, the pozzolanic reaction of CCGP, and the nucleation effect of cement hydration [27]. Given the effects of the pozzolanic reaction of CCGP and cement hydration and the need to meet the strength requirements while keeping the compressive strength difference minimal, a replacement rate of 30% was preferred.

### 3.2. HD-RRM Mixture Proportion Performance Optimization

#### 3.2.1. Effects of Water-to-Binder Ratio on the Compressive Strength of HD-RRM

**Figure 6:**
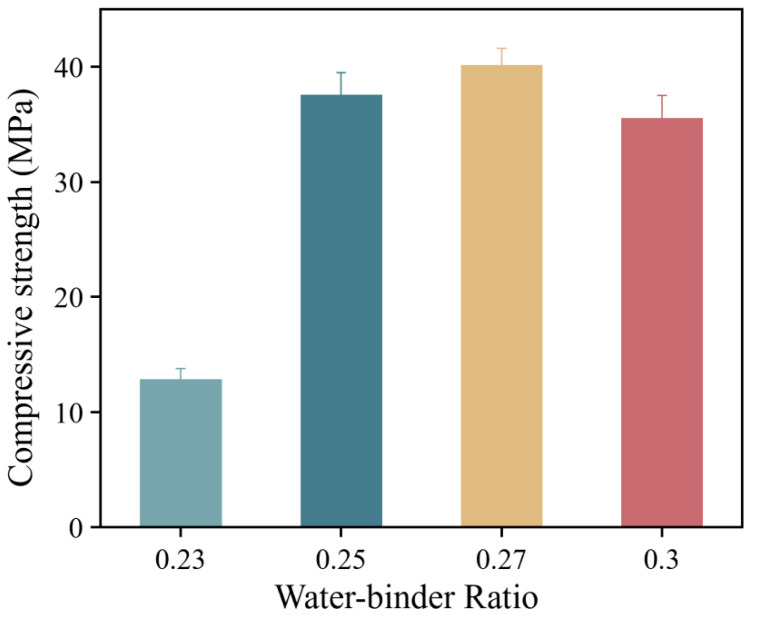
Compressive strengths of HD-RRM materials under different water-to-adhesive ratios.

The compressive strength and flexural strength of HD-RRM specimens with different water-to-binder ratios were investigated after 6 h of curing, and the results are presented in Figure 6. As depicted in Figure 6, at a curing time of 6 h, the compressive and flexural strengths of HD-RRM specimens first increased and then decreased with an increase in the water-to-binder ratio. When the water-to-binder ratio increased from 0.23 to 0.25, the compressive strength was enhanced by 213%. However, when the water-to-binder ratio increased from 0.25 to 0.30, the compressive strength declined by 11.5%. The optimal water-to-binder ratio for HD-RRM cubic specimens was 0.27.

With a lower water-to-binder ratio, the mixture exhibited poor flowability, leading to increased air voids in the matrix and a higher air content. Moreover, an extremely low water-to-binder ratio can cause PVA fibers to agglomerate and not disperse evenly, resulting in more defects in the specimens [28]. On the other hand, at a higher water-to-binder ratio, the fibers can be more significantly suspended on the surface during mixing, and increased water content can reduce the relative cement content, such that the material density and the bonding strength between the matrix and fibers can be reduced. Accordingly, the optimal water-to-binder ratio for HD-RRM was 0.27.

#### 3.2.2. Effects of Fiber Content on the Compressive Strength of HD-RRM

**Figure 7:**
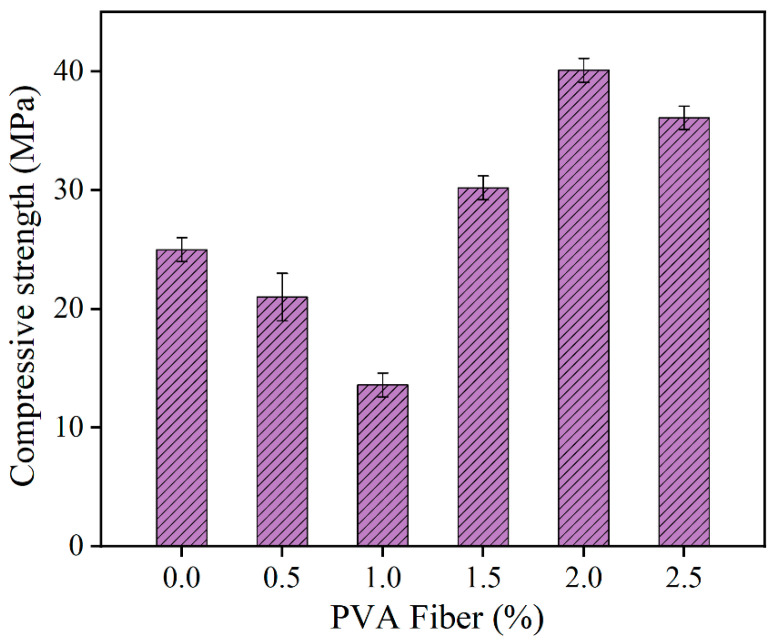
Compressive strengths of HD-RRM materials with different fiber dosages.

The compressive strength and flexural strength of HD-RRM specimens with different fiber contents were investigated after 6 h of curing, and the results are shown in Figure 7. As depicted in Figure 7, at a curing time of 6 h, the compressive strength of HD-RRM specimens first decreases, then increases, then decreases again with an increase in fiber content. When the fiber content increases from 0% to 1%, compressive strength decreases by 45.6%, and when the fiber content increases to 2%, compressive strength increases by 62.3%.

**Figure 8:**
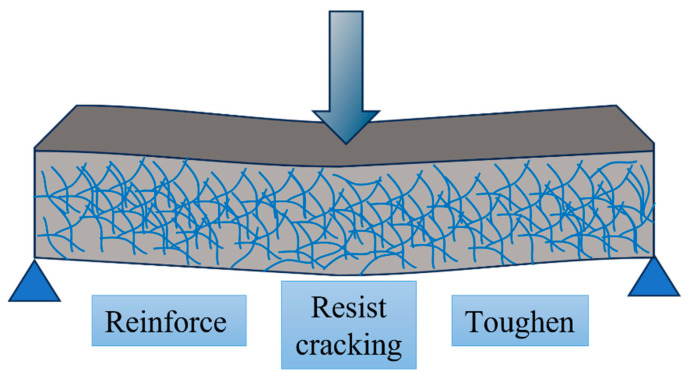
Mechanism of action of PVA fibers.

Concrete contains pores and defects, and, under external forces, stress concentration occurs in the abovementioned areas, leading to cracking and failure of the concrete. The addition of PVA fibers compensates for the internal defects of the concrete, obstructing the development of microcracks and exerting the bridging effect of PVA fibers, thereby increasing the strength of the matrix, as shown in Figure 8. When the fiber content is 1.0%, the compressive strength is lower than that of specimens without fibers. This is because the volume of PVA fibers is too low at this point, and they cannot exert their bridging enhancement effect, but instead increase internal microcracks, and, because the incorporation of PVA fibers has a certain air-entraining effect, it weakens the adhesion between cementitious materials and aggregates in concrete, thus reducing the strength of the composite material. When the fiber content is too high, fiber agglomeration and uneven distribution between the fibers are likely to occur, reducing the feasibility of the concrete and leading to more defects, such as air voids, in the specimens [28], which further reduces the strength of the composite material. For HD-RRM, the optimal volume of fibers is 2%. When the fiber dosage is reasonable, the bonding effect with the cement matrix is better, the specimen’s porosity is reduced, and compactness is improved, so that the strength of the specimen is improved, and the fiber in the matrix may have a certain hoop effect, which further increases the compressive strength of the specimen [28].

#### 3.2.3. Effects of Different Types and Dosages of Early Strength Agents on the Compressive Strength of HD-RRM

**Figure 9:**
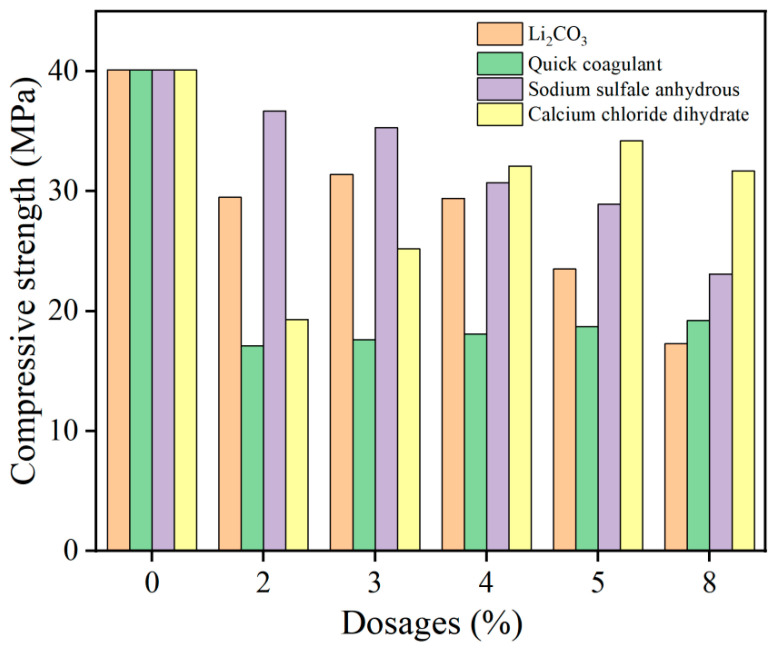
Compressive strengths of HD-RRM materials with different types of early strength agents and dosages.

The compressive strengths and flexural strengths of HD-RRM specimens with different types and dosages of early strength agents were investigated after 6 h of curing, and the results are shown in Figure 9. The compressive strengths of HD-RRM specimens with four types of early strength agents added were lower than that of the blank control group, showing no strength-enhancing effect; instead, it significantly decreased.

The cement used in the experiment is rapid-hardening sulfoaluminate cement, which hydrates rapidly. The addition of early strength agents accelerates cement hydration, leading to the formation of large crystals of ettringite, which do not disperse evenly in the slurry, resulting in areas with higher and lower hydration product levels, forming weak points that reduce strength and are more susceptible to damage [29]. On the other hand, during the material mixing process, Li_2_CO_3_ promotes cement hydration and releases considerable heat, causing the system temperature to rise excessively, leading to premature setting of the mixture. Incomplete hydration reactions in cementitious materials in the system lead to a decrease in material strength [30].

#### 3.2.4. Effects of Mineral Admixtures on Compressive Strength of HD-RRM

**Figure 10:**
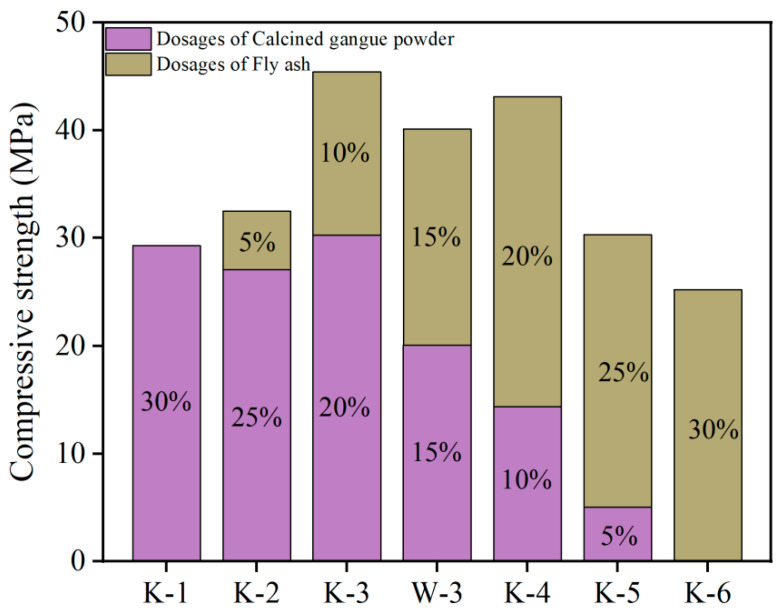
The influence of mineral admixtures on the compressive strength of HD-RRM materials.

Figure 10 shows that the compressive strength of cubic specimens with both CCGP and FA is higher than that of specimens with either of them alone. Among the cubic specimens with admixtures, three proportions achieved a compressive strength of 40 MPa, meeting the requirement of HD-RRM for the compressive strength of cubes. Among these, the K-3 proportion exhibited the maximum compressive strength, reaching 45.4 MPa.

**Figure 11:**
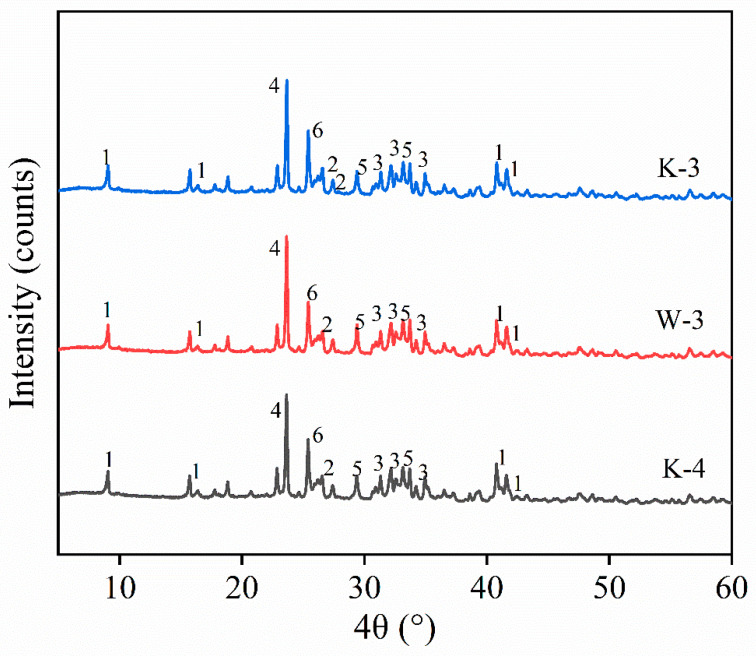
XRD plots of HD-RRM with different ratios: 1. Aft, 2. CaCO_3_, 3. C_3_S, 4. 3CaO·3Al_2_O_3_·CaSO_4_, 5. C_2_S, 6. Ca(OH)_2_.

XRD experiments were performed to explore the underlying mechanisms of the three proportions achieving a compressive strength of 40 MPa, with the results presented in Figure 11. As depicted in Figure 11, the three samples displayed similar XRD diffraction peaks, suggesting that the types of hydration products for the three proportions were nearly identical. To be specific, ettringite served as the main hydration product. At nearly 23°, a distinct diffraction peak of anhydrous calcium sulfoaluminate was observed. At 25°, a diffraction peak of Ca(OH)_2_ was reported. Anhydrous calcium sulfoaluminate acted as a major component of SAC cement, and its hydration led to the synthesis of Ca(OH)_2_. As indicated by comparison of the diffraction peaks of anhydrous calcium sulfoaluminate and Ca(OH)_2_ in terms of the three proportions, anhydrous calcium sulfoaluminate for the K-3 proportion achieved the weakest diffraction peak, whereas Ca(OH)_2_ displayed the most significant diffraction peak, suggesting that its hydration reaction achieved the greatest significance, such that the maximum compressive strength was achieved. The W-3 proportion displayed the most significant diffraction peak for anhydrous calcium sulfoaluminate and the weakest diffraction peak for Ca(OH)_2_, suggesting the minimum degree of hydration and the minimum compressive strength.

**Figure 12:**
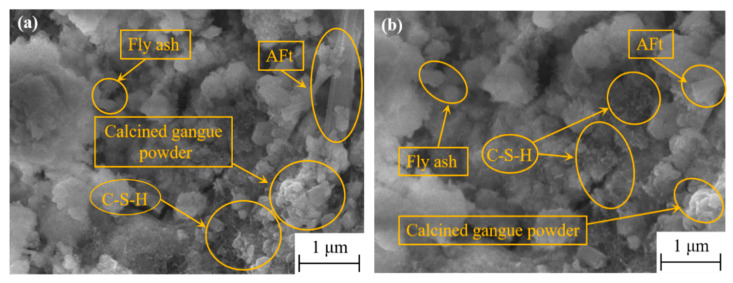

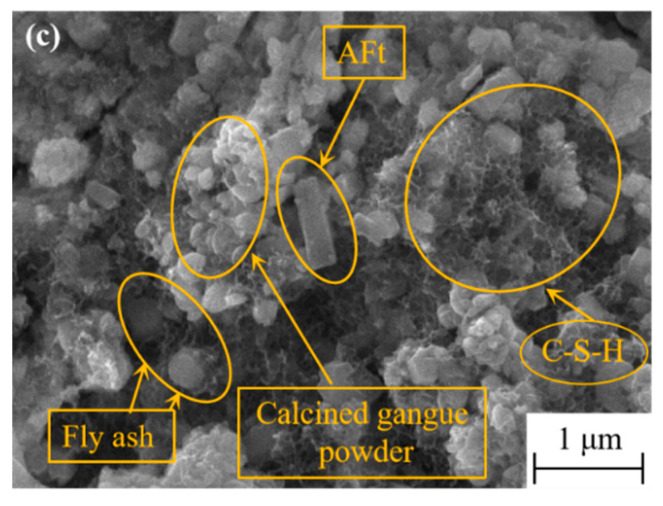
SEM diagram with different mix ratios: (**a**) K-4; (**b**) W-3; (**c**) K-3.

Figure 12 illustrates the morphology and microstructure of hardened cement paste in terms of K-4, W-3, and K-3 proportions after 6 h of standard curing. As depicted in Figure 12, the cement–coal gangue powder system comprised needle-like ettringite, cellular C-S-H gel, partially unreacted coal gangue powder, and FA particles. The early strength of the cement–coal gangue powder system was enhanced by the early formation of considerable ettringite, whereas the formation of cellular C-S-H gel endowed the cement–coal gangue powder system with a more compact internal structure, such that the later strength growth of the system was ensured. As depicted in Figure 12, some C-S-H gel adhered to the surface of the coal gangue powder and FA particles for the prominent pozzolanic properties of CCGP and FA [31], and they were subjected to secondary hydration in the alkaline environment provided by cement hydration, such that C-S-H gel that wrapped the surface was generated [32].

**Figure 13:**
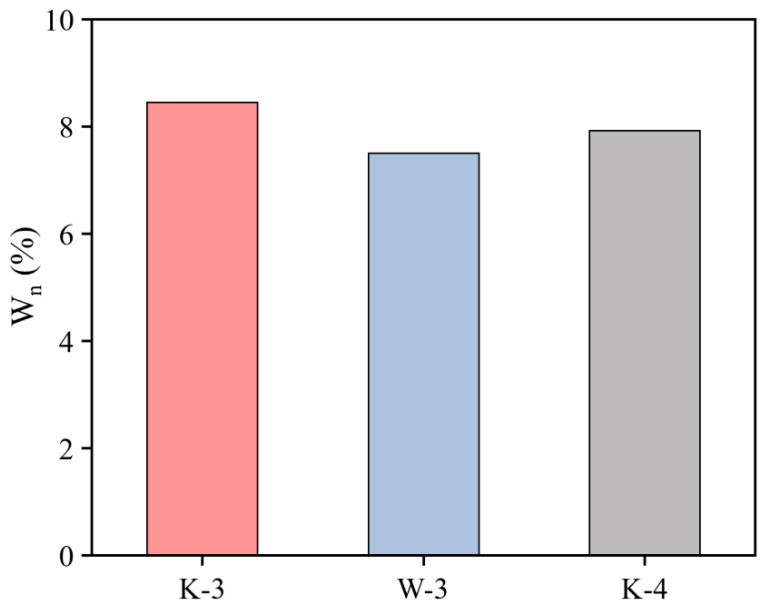
Non-evaporative water content diagram with different mixing ratios.

**Figure 14:**
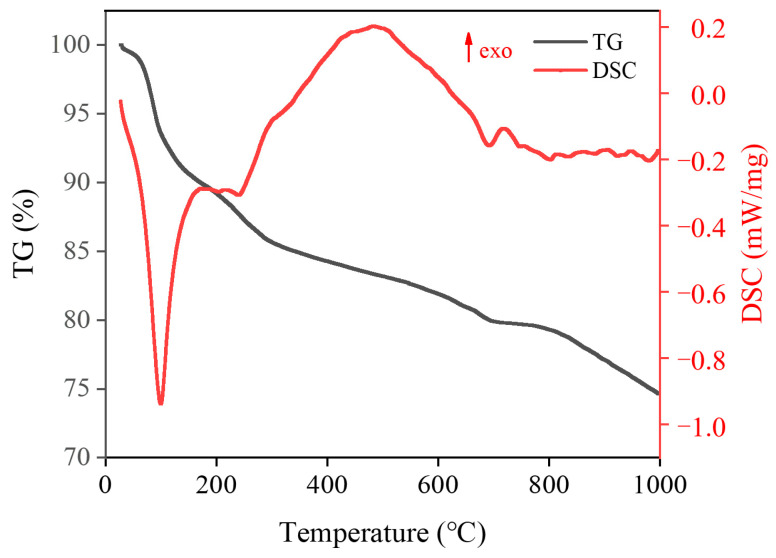
TG-DSC curve for K-3.

Additionally, as depicted in Table 7 and Figure 13, the non-evaporable water content of the K-3 proportion exceeded that of the other two proportions. In general, the non-evaporable water was chemically bound water in hydration products, comprising interlayer water in C-S-H gel as well as crystalline water in alkali-silicate calcium aluminate hydrate (AFm) and alkali-ferric calcium aluminate hydrate (AFt). In cement-based composite systems with mineral admixtures, the non-evaporable water content indicated the quantity of hydration products while indirectly suggesting the degree of hydration of the cement and mineral admixtures [33], thus clarifying that the K-3 proportion exhibited the maximum degree of hydration and, therefore, the maximum strength. TG-DSC experiments were performed to investigate the hydration of the K-3 proportion in depth, and the results are presented in Figure 14. A wide range of hydration products decomposed or lost water at different temperatures in the heating process for cementitious material hardened paste specimens. Most of the bound water in C-S-H gel evaporated between 100 °C and 400 °C, and Ca(OH)_2_ decomposed and lost water between 400 °C and 550 °C. Some CaCO_3_ decomposed around 600 °C to 750 °C. Furthermore, AFm generated from aluminum, iron, sulfur, and other phases during hydration contained a small amount of bound water, decomposing between 100 °C and 400 °C [34,35]. As indicated by the TG-DSC curve, the first endothermic peak, where the mass declined significantly from 65 °C to 200 °C, primarily belonged to the endothermic evaporation of adsorbed water in the C-S-H gel and coordinating water in AFt. The second endothermic peak belonged to the evaporation of interlayer water in the C-S-H gel and crystalline water in ettringite. The third endothermic peak belonged to the complete loss of bound water in ettringite, which decomposed into Al_2_O_3_ and CaO, whereas C-S-H gel lost considerable interlayer water while decomposing and breaking down. At a temperature approaching 900 °C, smaller endothermic and exothermic peaks were identified, belonging to the phase transitions of cementitious materials between 800 °C and 900 °C. As a result, exothermic and endothermic effects were generated, but no change in mass was reported.

In brief, the preferred mixture proportions for the experiment were a water-to-binder ratio of 0.27, a sand-to-binder ratio of 0.3, a fiber volume content of 2%, a cement content of 70%, a CCGP content of 20%, a FA content of 10%, and a superplasticizer dosage of 0.1%. With the above-described proportions, the composite material, after 6 h of standard curing, achieved a compressive strength of 45.4 MPa, meeting the strength design specifications of C40 for cube compressive strength at an early time of 6 h.

#### 3.2.5. Tensile Performance of HD-RRM

**Figure 15:**
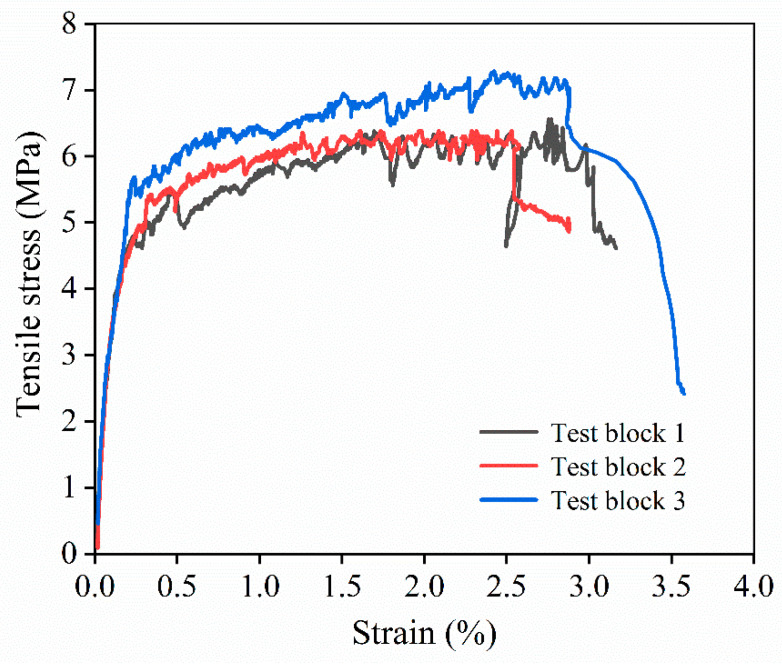
HD-RRM uniaxial tensile stress–strain relationship curve.

In accordance with the compressive strength test results for the cubic specimens, K-3’s proportions were identified as optimal. Tensile specimens were prepared using the above-described optimal proportions for tensile performance testing. The stress–strain relationship curve is illustrated in Figure 15, and Table 8 lists the characteristic parameters. As depicted in the above figure and table, the stress–strain curve of HD-RRM mainly had three stages, as follows. The first stage was the linear elastic stage, where stress and strain developed proportionally until the first crack appeared. The second stage was the strain-hardening stage, during which multiple cracks occurred in the material. The third stage was the strain-softening stage, where cracks further widened and extended till the specimen fractured [36]. The peak stress *σ*_t,u_ and peak strain *ε*_t,u_ represent the stress and strain values at the peak points, respectively. The initial cracking tensile strength *σ*_t,*f*_ expresses the stress value at the critical point where the stress–strain curve departs from the initial linear elastic segment. The initial cracking elongation *ε*_t,*f*_ represents the strain at the initial cracking stress, and the tensile elastic modulus *E*_t_ represents the slope of the initial linear elastic segment of the stress–strain curve. As indicated by the data in the table, the ultimate elongation of this proportion was 2.75%, greater than the required minimum of 2%, and the average crack width reached 125.53 μm, smaller than the maximum allowable value of 200 μm, thus conforming to the material development target. The slag-based repair material prepared by Li Ming et al. [37] had a 3d compressive strength of 24.1 MPa; the solid waste geopolymer pavement repair material prepared by Song Luxia et al. [38] had a 3d compressive strength of 53.48 MPa. Compared to them, HD-RRM had a shorter curing time and a better compressive strength, and they did not take into account ultimate tensile strength or ultimate elongation.

**Figure 16:**
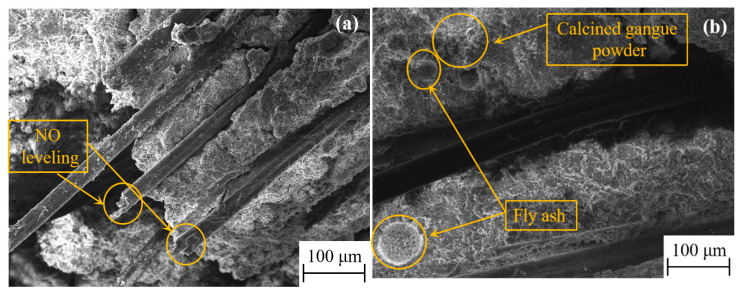
Diagram of PVA fiber SEM in the HD-RRM material: (**a**) Fiber cross section; (**b**) Effect of mineral admixtures on fibers.

The high ductility of HD-RRM is largely dependent on the bridging effect between the PVA fibers and the matrix. Under a weak bond strength between the fibers and the matrix, stress cannot be effectively transferred from the matrix to the fibers, and the fibers cannot facilitate effective toughening. On the other hand, under excessively high bond strength, fiber brittle fractures will be triggered, such that the achievement of strain-hardening characteristics can be hindered, and the toughening effect of PVA fibers will not be evident [39]. Therefore, the toughening effect of the fibers will be significant only when the bond between the PVA fibers and the matrix reaches an ideal state. As shown in Figure 16a, the cross-sectional surface of PVA fibers is uneven, suggesting that fibers were pulled out during the experiment rather than being broken, fully demonstrating the toughening effect of the fibers. This is because, after the complex addition of CCGP and FA, the matrix and the interface contain unhydrated or partially hydrated particles of CCGP and FA (Figure 16b). The abovementioned particles reduce the friction and scraping effects of hydrated products on the fibers, which is beneficial to the toughening effect of PVA fibers. Additionally, because CCGP has good pozzolanic properties, it not only facilitates the absorption of calcium hydroxide during cement hydration but also reacts with it to form hydrated calcium silicate gel, making the matrix material more uniform and avoiding crack concentration.

## 4. Conclusions

(1) As indicated by the activity test of CCGP through the strength evaluation method, the smaller the particle size of CCGP, the higher the strength of the mortar specimens would bel the optimal particle size was 1250 mesh, suggesting the maximum potential activity. With an increase in the content of CCGP, the strength of the mortar specimens declines. Given the strength and utilization of CCGP, the optimal content of CCGP (mineral admixture) was determined to be 30%.
(2) The degrees of hydration and the types of hydration products for the K-4, W-3 and K-3 ratios are basically the same; no new substances are produced, and the main hydration products are AFt and C-S-H gel. It can be seen that the main role in the hydration process for HD-RRM materials is played by SAC; calcined gangue powder and fly ash have a lower degree of hydration, and mainly play the role of physical filling.
(3) After studying the influences of water–cement ratio, fibers, early strength agents, and mineral admixture on the strength of HD-RRM, the optimal proportions were a water-to-binder ratio of 0.27, a sand-to-binder ratio of 0.3, a PVA fiber content of 2%, a SAC content of 70%, a mineral admixture content of 30% (20% CCGP and 10% FA), and a superplasticizer dosage of 0.1%. With the abovementioned proportions, after 6 h of standard curing, HD-RRM was endowed with an ultimate elongation of 2.75%, an ultimate tensile strength of 7.58 MPa, a compressive strength of 45.4 MPa, and an average crack width of 125.53 μm.

## Figures and Tables

**Table 1:** Chemical composition of SAC and CCGP of different particle sizes/%.

Chemical Composition	Al_2_O_3_	SiO_2_	CaO	Fe_2_O_3_	MgO	K_2_O	SO_3_	Loss
SAC	23.09	10.50	43.99	2.46	1.54	0.33	13.20	~
300-mesh	13.71	37.77	13.17	18.83	5.32	3.41	0.19	4.33
325-mesh	46.40	50.16	0.27	0.51	~	0.15	0.03	3.87
600-mesh	45.90	50.74	0.25	0.46	~	0.11	0.03	3.32
800-mesh	13.35	38.59	11.47	18.73	6.98	3.25	0.15	4.21
1250-mesh	47.23	48.28	0.32	0.79	~	0.21	0.04	4.14

**Table 2:** Main properties of PVA fibers.

Diameter (μm)	Length (mm)	Density (kg∙m^−3^)	Elastic Modulus (GPa)	Ultimate Tensile Strength (MPa)	Ultimate Elongation (%)
39	12	1300	30	≥1250	5~8

**Table 3:** Composition designs of HD-RRM with different water–binder ratios.

Number	SAC	CCGP	FA	Sand	Water	PC	Fibers
A1	0.7	0.15	0.15	0.3	0.23	0.001	2%
A2	0.7	0.15	0.15	0.3	0.25	0.001	2%
A3	0.7	0.15	0.15	0.3	0.27	0.001	2%
A4	0.7	0.15	0.15	0.3	0.30	0.001	2%

**Table 4:** Composition designs of HD-RRM with different fiber contents.

Number	SAC	CCGP	FA	Sand	Water	PC	Fibers
B1	0.7	0.15	0.15	0.3	0.25	0.001	1%
B2	0.7	0.15	0.15	0.3	0.25	0.001	1.5%
B3	0.7	0.15	0.15	0.3	0.25	0.001	2%
B4	0.7	0.15	0.15	0.3	0.25	0.001	2.5%

**Table 5:** Composition designs of HD-RRM with different early strength agents and their dosages.

No.	SAC	CCGP	FA	S	W	PC	Li_2_CO_3_ (%)	QC (%)	SSA (%)	CCD (%)	Fibers
C1	0.7	0.15	0.15	0.3	0.27	0.001	2	0	0	0	2%
C2	0.7	0.15	0.15	0.3	0.27	0.001	3	0	0	0	2%
C3	0.7	0.15	0.15	0.3	0.27	0.001	4	0	0	0	2%
C4	0.7	0.15	0.15	0.3	0.27	0.001	5	0	0	0	2%
C5	0.7	0.15	0.15	0.3	0.27	0.001	8	0	0	0	2%
C6	0.7	0.15	0.15	0.3	0.27	0.001	0	2	0	0	2%
C7	0.7	0.15	0.15	0.3	0.27	0.001	0	3	0	0	2%
C8	0.7	0.15	0.15	0.3	0.27	0.001	0	4	0	0	2%
C9	0.7	0.15	0.15	0.3	0.27	0.001	0	5	0	0	2%
C10	0.7	0.15	0.15	0.3	0.27	0.001	0	8	0	0	2%
C11	0.7	0.15	0.15	0.3	0.27	0.001	0	0	2	0	2%
C12	0.7	0.15	0.15	0.3	0.27	0.001	0	0	3	0	2%
C13	0.7	0.15	0.15	0.3	0.27	0.001	0	0	4	0	2%
C14	0.7	0.15	0.15	0.3	0.27	0.001	0	0	5	0	2%
C15	0.7	0.15	0.15	0.3	0.27	0.001	0	0	8	0	2%
C16	0.7	0.15	0.15	0.3	0.27	0.001	0	0	0	2	2%
C17	0.7	0.15	0.15	0.3	0.27	0.001	0	0	0	3	2%
C18	0.7	0.15	0.15	0.3	0.27	0.001	0	0	0	4	2%
C19	0.7	0.15	0.15	0.3	0.27	0.001	0	0	0	5	2%
C20	0.7	0.15	0.15	0.3	0.27	0.001	0	0	0	8	2%

**Table 6:** Composition designs of HD-RRM with different mineral admixtures.

Number	SAC	CCGP	FA	Sand	Water	PC	Fibers
D1	0.7	0.3	0	0.3	0.27	0.001	2%
D2	0.7	0.25	0.05	0.3	0.27	0.001	2%
D3	0.7	0.2	0.1	0.3	0.27	0.001	2%
D4	0.7	0.15	0.15	0.3	0.27	0.001	2%
D5	0.7	0.1	0.2	0.3	0.27	0.001	2%
D6	0.7	0.05	0.25	0.3	0.27	0.001	2%
D7	0.7	0	0.3	0.3	0.27	0.001	2%

**Table 7:** Test data for non-evaporated water content with different mixing ratios.

Age	Number	Sample Mass after Drying m_0_ (g)	Non-Evaporated Water Mass m_n_ (g)	Non-Evaporated Water Content Wn (%)	Loss on Burning of Cementitious Materials Lc (%)
6 h	K-3	1.0013	0.8789	8.45	4.14
	W-3	1.0011	0.8894	7.50	
	K-4	1.0016	0.8843	7.92	

**Table 8:** Characteristic parameters in the tensile test.

σ_t,f_ (MPa)	ε_t,f_ (%)	E_t_ (GPa)	σ_t,u_ (MPa)	ε_t,u_ (%)	The Average Crack Width (μm)
2.98 ± 0.062	0.235 ± 0.001	12.7 ± 0.200	7.58 ± 0.183	2.75 ± 0.070	125.53 μm

## Data Availability

The data presented in this study are available on request from the corresponding author.

## References

[1] Lu K.Y. (2016). Analysis and prospect of comprehensive utilization of coal solid waste. Coal Process. Compr. Util..

[2] Wang A., Liu P., Mo L., Liu K., Ma R., Guan Y., Sun D. (2022). Mechanism of thermal activation on granular coal gangue and its impact on the performance of cement mortars. J. Build. Eng..

[3] Chen J., Shui Z.H., Sun T., Gao X., Song Q., Guo C. (2019). Early hydration kinetics research of calcined coal gangue in cement-based materials. Bull. Chin. Ceram. Soc..

[4] Yang J., Su Y., He X., Tan H., Jiang Y., Zeng L., Strnadel B. (2018). Pore structure evaluation of cementing composites blended with coal by-products: Calcined coal gangue and coal fly ash. Fuel Process. Technol..

[5] Zhou M., Qu H.L., Zhao H.M. (2015). Effect of Calcined Coal Gangue Powder on Workability and Strength of Concrete. Bull. Chin. Ceram. Soc..

[6] Wang Y., Hou M., Yu J., Xu S., Yu K., Zhang Z. (2018). Experimental study on mechanical properties of ultra-high ductile cementitious composites. Mater. Rep..

[7] Li-li K.A.N., Zhi Z., Li Z., Wei-dong L.I.U. (2020). A dynamic constitutive model of ultra high toughness cementitious composites. J. Zhe Jiang Univ. Sci..

[8] Kan L.L., Zhang Z., Zhang L., Wei-dong L. (2019). Effect of low-cost PVA fibers on the mechanical properties of engineered cementitious composites. Eng. Mech..

[9] Smail M.K., Sherir M.A., Siad H., Hassan A.A., Lachemi M. (2018). Properties of self-consolidating engineered cementitious composite modified with rubber. J. Mater. Civ. Eng..

[10] Yuan F., Chen M.C., Pan J.H. (2020). Flexural strengthening of reinforced concrete beams with high-strength steel wire and engineered cementitious composites. Constr. Build. Mater..

[11] Guo L.P., Yang Y.N., Chen B. (2019). A uniaxial tensile constitutive equation of high-ductility cementitious composites. J. Southeast Univ..

[12] Guo L.P., Wang M., Ding C., Cai L.J. (2020). Effect of incorporating reclaimed asphalt pavement on macroscopic and microstructural properties of high ductility cementitious composites. Constr. Build. Mater..

[13] Li J.X., Weng J., Yang E.H. (2019). Stochastic model of tensile behavior of strain-hardening cementitious composites (SHCCs). Cem. Concr. Res..

[14] Wang Z.B., Zhang J., Wang Q. (2018). Mechanical behavior and crack width control of hybrid fiber reinforced ductile cementitious composites. J. Build. Mater..

[15] Zhu H., Yu K., Li V.C. (2021). Sprayable engineered cementitious composites (ECC) using calcined clay limestone cement (LC3) and PP fiber. Cem. Concr. Compos..

[16] Feng H., Chen G., Hadi M.N., Sheikh M.N., Zhou B. (2018). Mechanical behaviour of micro-fine steel fibre reinforced sulphoaluminate cement composite. Constr. Build. Mater..

[17] Lv L., Hong X., Ding Z., Ma X., Li H. (2020). Preparation and characterization of calcium sulfoaluminate based engineered cementitious composites for rapid repairing of concrete member. Front. Mater..

[18] (2017). Technical Specifications of Cement Concrete Pavement Maintenance for Highway.

[19] (2011). Specification of Cement Concrete Pavement Design for Highway.

[20] (2006). Sulphoaluminate Cement.

[21] Chai L.J., Guo L.P., Chen B., Xu Y.H. (2018). Interactive effects of freeze-thaw cycle and carbonation on tensile property of ecological high ductility cementitious composites for bridge deck link slab. Constr. Build. Mater..

[22] (2020). Method of Testing Cements—Determination of Strength.

[23] (2017). Fly Ash Used for Cement and Concrete.

[24] (2018). Standard Test Method for the Mechanical Properties of Ductile Fiber Reinforced Cementitious Composites.

[25] Zhao H.S., Zhang X., Cao J. (2004). Grey related analysis on the relationship between the particle size distribution of coal gangue and its activity. Cem. Eng..

[26] Wei J., Gencturk B., Jain A., Hanifehzadeh M. (2019). Mitigating alkali-silica reaction induced concrete degradation through cement substitution by metakaolin and bentonite. Appl. Clay Sci..

[27] Li W., Hua L., Shi Y., Wang P., Liu Z., Cui D., Sun X. (2022). Influence of metakaolin on the hydration and microstructure evolution of cement paste during the early stage. Appl. Clay Sci..

[28] Zhang Y.T., Zhang Y.S., Wu Z.T., Liu N.D., Yuan T.F. (2019). Ptimization Design and Properties of Glass Fiber Reinforced Cementitious Composities. Mater. Rev..

[29] Han J.G., Yan P.Y. (2011). Influence of Lithium Carbonate on Hydration Characteristics and Strength Development of Suiphoaluminate Cement. J. Build. Mater..

[30] Chen T., Ren B., Wang Z., Meng X., Ning Y., Lv Y. (2022). Effect of early strength agent on the hydration of geopolymer mortar at low temperatures. Case Stud. Constr. Mater..

[31] Rafieizonooz M., Mirza J., Salim M.R., Hussin M.W., Khankhaje E. (2016). Investigation of coal bottom ash and fly ash in concrete as replacement for sand and cement. Constr. Build. Mater..

[32] Huang H., Guo M.X., Zhang W. (2022). Mechanical property and microstructure of geopolymer concrete based on fly ash and slag. J. Harbin Inst. Technol..

[33] Jia Y.T. (2005). Study on hydration mechanism of slag and fly ash cement-based materials. Master’s Thesis.

[34] El-Jazairi B., Illston J.M. (1977). A simultaneous semi-isothermal method of thermogravimetry and derivative thermogravimetry, and its application to cement pastes. Cem. Concr. Res..

[35] Taylor H.F.W., Mohan K., Moir G.K. (1985). Analytical Study of Pure and Extended Portlan Cement Pastes: II, Fly Ash-and Slag-Cement Pastes. J. Am. Ceram. Soc..

[36] Kanda T., Lin Z., Li V.C. (2011). Tensile stress-strain modeline of pseudo strain hardening cementitious composites. J. Mater. Civ. Eng..

[37] Li M., Zhi X.L., Yao A.L. (2014). Study on Slag Based Cement Concrete Pavement Repair Material. Mater. Rev..

[38] Song L.X., Hao X.F., Duan P., Huang M., Zhang H. (2022). Preparation of Solid Waste Geopolymer Pavement Repairing Material and Performance Study. J. Hubei Univ. Educ..

[39] Wang H.L., Luo Y.J., Peng G.Y., Sun X., Ying Q. (2017). Effect of Admixtures on Tensile Behavior of Fiber Reinfbrced Cementitious Composites. J. Southwest Jiao Tong Univ..

